# Recombinant Adiponectin Ameliorates Liver Ischemia Reperfusion Injury via Activating the AMPK/eNOS Pathway

**DOI:** 10.1371/journal.pone.0066382

**Published:** 2013-06-07

**Authors:** Chuanzhao Zhang, Yuan Liao, Qiang Li, Maogen Chen, Qiang Zhao, Ronghai Deng, Chenglin Wu, Anli Yang, Zhiyong Guo, Dongping Wang, Xiaoshun He

**Affiliations:** Organ Transplant Center, the First Affiliated Hospital, Sun Yat-sen University, Guangzhou, China; UAE University, Faculty of Medicine & Health Sciences, United Arab Emirates

## Abstract

**Background:**

It is of importance to minimize ischemia reperfusion (I/R) injury during liver operations. Reducing the inflammatory reaction is an effective way to achieve this goal. Notably, adiponectin (APN) was found to have anti-inflammatory activity in heart and renal I/R injury. Herein, we investigated the role of APN in liver I/R injury.

**Methods:**

Wistar rats were randomized to four groups: (1) sham group; (2) I/R control group; (3) I/R+APN group; and (4) I/R+APN+AMPK inhibitor group. Liver and blood samples were collected 6h and 24h after reperfusion. Liver function and histopathologic changes were assessed. Macrophage and neutrophil infiltration was detected by immunohistochemistry staining, while pro-inflammatory cytokines and chemokines released in the liver were measured using ELISA and RT-PCR, respectively. Apoptosis was analyzed by TUNEL staining and caspase-3 expression in the liver. Downstream molecules of APN were investigated by Western blotting.

**Results:**

Circulatory APN was down-regulated during liver I/R. When exogenous APN treatment was administered during liver I/R, alanine transaminase (ALT) and aspartate aminotransferase (AST) were decreased, and less hepatocyte necrosis was observed. Less inflammatory cell infiltration and pro-inflammatory cytokines/chemokines release were also observed in the I/R+APN group when compared with the I/R control group. APN treatment also reduced hepatocyte apoptosis, evidenced by reduced TUNEL positive cells and less caspase-3 expression in the reperfused liver. Finally, the AMPK/eNOS pathway was found to be activated by APN, and administration of an AMPK inhibitor reversed the beneficial effects of APN.

**Conclusion:**

APN can protect the liver from I/R injury by reducing the inflammatory response and hepatocyte apoptosis, a process that likely involves the AMPK/eNOS pathway. The current study provides a potential pharmacologic target for liver I/R injury.

## Introduction

Liver ischemia reperfusion (I/R) injury is still a critical problem that affects patient outcomes after liver resection and transplantation. Both the inflammatory response and hepatocyte apoptosis contribute to I/R injury, resulting in significant cellular damage and organ dysfunction[1], [2]. Multiple cellular and molecular pathways have been proven to be involved in the pathogenesis of liver I/R. For instance, Toll-like receptor 4 (TLR4)/interferon regulatory factor (IRF3) signaling was involved in the process of liver I/R injury[3], while Wnt-β-catenin signaling regulated hepatocellular responses to liver I/R injury[4]. In addition, Peralta C et al. demonstrated that adenosine monophosphate-activated protein kinase (AMPK) activation could reduce liver I/R injury[5]. However, the substances that activate these pathways in vivo are still far from clear.

Adiponectin (APN), also called gelatin-binding protein-28 (GBP28), AdipoQ, ACRP30 or apM1, is a secreted hormone derived from adipocytes. It was considered first as an important regulator of energy use and metabolism in endocrinology[6]. Recently, more attention has been paid to the anti-inflammatory and anti-apoptotic properties of APN[7], [8]. One of the mechanisms through which APN acts is to induce activation of AMPK/endothelial nitric oxide synthase (eNOS) signaling[6], [9], [10]. Shibata et al. have shown that cardiac infarct size and cell apoptosis were increased in APN-KO mice in an I/R model[11]. Similarly, a beneficial outcome was observed in cerebral I/R mice treated with APN[12], [13]. Although there is mounting evidence showing the role of APN in other organ I/R injury, whether APN is involved in liver I/R injury is unclear. In a recent study, a combined APN and FTY720 therapy was used in a small-for-size fatty liver transplant model, and this treatment significantly improved liver graft survival[14]. This work indicates a possible protective activity of APN in fatty liver suffering I/R injury. However, the effects of APN on non-fatty I/R liver remain to be elucidated.

In this study, we show that serum APN is down-regulated after liver I/R. In addition, exogenous APN treatment may ameliorate liver I/R injury by preventing inflammatory cell infiltration, reducing pro-inflammatory cytokines/chemokines secretion, and reducing hepatocyte apoptosis. We also demonstrate that the protective role of APN in liver I/R injury is dependent on AMPK activation. This work might provide insights into the development of a novel strategy to treat liver I/R injury.

## Materials and Methods

### Animals

A total of 80 healthy male Wister rats (purchased from the Animal Center of Sun Yat-sen University, Guangzhou, China), weighing 200–250 g, were maintained in a specific pathogen-free environment in our facility. All animals were fed with standard chow and had free access to water. All animal experiments were performed in a humane manner, and also in accordance with the Institutional Animal Care Instructions. This study was conducted under experimental protocols approved by the Ethics Committee for Animals, Sun Yat-sen University (approval ID: 2010 NO.9).

### Liver I/R model and experiment design

The animals were randomly divided into four groups (10 rats in each group): (1) sham group, the rats underwent anesthesia, laparotomy, and exposure of the portal triad but without hepatic ischemia; (2) I/R control (I/R ctrl) group, the rats were subjected to I/R and treated with PBS during reperfusion; (3) I/R+APN group, the rats were subjected to I/R and treated with APN (0.5 mg/kg, diluted in PBS) during reperfusion; (4) I/R+APN+8-bromo-AMP (bAMP) group, the rats were treated with an AMPK inhibitor, bAMP, before ischemia, and APN was used during reperfusion. bAMP was diluted in PBS at a concentration of 5 mmol/L, and was infused at a dose of 0.1 mL/kg/min intravenously. A 70% liver I/R model was established in the latter three groups, as described previously[15]. In brief, the portal vein, the hepatic artery, and the bile duct supplying the median and the left lateral lobes of the liver were clamped with a microvessel clip. After 60 min ischemia, the clip was removed and reperfusion started. At the indicated time points (6h and 24h after reperfusion), the rats were sacrificed and the injured liver tissue and blood samples were collected for analysis.

### Liver function tests

Rats were sacrificed and blood samples were collected from the abdominal aorta. After remaining still for one hour in room temperature, the blood was centrifugated for 10 minutes and the serum was separated. Blood serum was analyzed for aspartate aminotransferase (AST) and alanine aminotransferase (ALT) using methods as described previously[16].

### Histopathological and immunohistochemical studies

All liver samples were fixed in 10% buffered formalin overnight, embedded in paraffin and sectioned. Sections (5μm) were stained with hemotoxylin and eosin for histological analyses. In the histopathological studies, the severity of liver IRI (necrosis, sinusoidal congestion, and centrilobular ballooning) was blindly graded with modified Suzuki's criteria on a scale from 0–4[17]. In immunohistochemical studies, CD68 and MPO were used as macrophage and neutrophil markers to detect inflammatory cells infiltration. The primary antibody was a rabbit monoclonal anti-mouse/rat/human CD68 or MPO antibody (Cell Signaling, MA, USA). Immunohistochemistry was performed using a biotin-free enhanced polymer one-step staining technique (EPOS-method) according to the manufacture’s guidelines with a peroxidase-conjugated polymer backbone coupled with a goat anti-rabbit secondary antibody (Cell Signaling, MA, USA). Liver sections were evaluated blindly by counting labeled cells in 10 high-power fields (HPF).

### Terminal deoxynucleotidyl transferase-mediated dUTP nick-end labeling (TUNEL) assay

Apoptosis in liver tissues was identified by TUNEL assay with an in situ Cell Death Detection kit (Roche Applied Science, IN, USA) following the manufacturer's instruction[18]. Quantitative analysis was presented as percentage of TUNEL-positive hepatocyte nuclei per total nuclei in each experimental group.

### Expression and Purification of Recombinant Adiponectin

Rat adiponectin (encoding residues 20-244, without the secretory leader peptide sequence) were cloned into the pET30 vector (Novagen, Darmstadt, Germany). DNA constructs were transformed into BL21 (DE3) *Escherichia coli* (Novagen, Darmstadt, Germany). The expression of His-tagged adiponectin was induced with isopropyl-1-thio-β-D-galactopyranoside at 37°C. Recombinant His-tagged fusion protein was isolated from the cytoplasm and purified with a His·Bind resin column (Novagen, Darmstadt, Germany).

### Western blot analyses

Western blotting was carried out as described previously[19]. The antibodies used here were anti-caspase 3, anti-AMPK, anti-pAMPK, anti-eNOS, anti-peNOS and anti-GAPDH antibodies (Cell Signaling, MA, USA).

### Real-time Polymerase Chain Reaction

Total RNA was extracted from liver using TRIzol Reagent (Invitrogen, Carlsbad, CA) and cDNA was synthesized using the High Capacity RNA-to-cDNA Kit (Applied Biosystems, Foster, CA) according to the manufacturer’s instructions. Real-time polymerase chain reaction (PCR) was performed using SYBR® Premix Ex Taq™ II (Takara, Dalian, China) on the 7900HT Fast Real-Time PCR System (Applied Biosystems, Foster, CA). Murine β-actin (F:5′-AAGTGCTTCTAGGCGG

ACTGTT-3′ R:5′-TTTTCTGCGCAAGTTAGGTTTTG-3′) was used as an internal control. The primers used were as follow: CCL-2(F: 5′-ATGCAGTTAATGCCCCA

CTC-3′, R: 5′-TTCCTTATTGGGGTCAGCAC-3′), CXCL-10(F: 5′-GGGCCATAG

GAAAAC TTGAAATC-3′, R: 5′-CATTGTGGCAATGATCTCAACAT-3′) and ICAM-1(F: 5′-AAACGGGAGATGAATGGTACCTAC-3′, R: 5′-TGCACGTCCC

TGGTGATACTC-3′) (Invitrogen, Carlsbad, CA).

### Serum APN measurement

Serum APN concentration was assessed by rat APN ELISA kit (R&D Corp, MN, USA) according to the manufacturer’s protocols.

### Liver tissue cytokines measurement

Liver samples were homogenized and centrifuged to get the liquid supernatant. The IL-1β, IL-6, and TNF-α concentration of the liquid supernatant were measured by an enzyme linked immunosorbent assay (ELISA) technique as previously reported using the commercial kit (USCN, Wuhan, China)[20].

### Statistical analysis

All data are expressed as mean ± standard deviation. The statistical analysis was performed by one-way analysis of variance (ANOVA). A P value of less than 0.05 was considered to be statistically significant.

## Results

### Dynamic change of serum APN concentration after liver I/R

**Figure 1:**
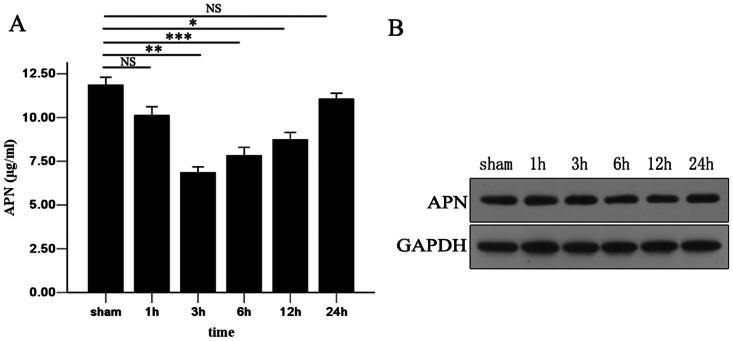
Dynamic change of serum APN concentration after liver I/R. (A) Serum APN concentration at 1h, 3h, 6h, 12h, and 24h after reperfusion were assessed by ELISA. (B) Expression of APN in liver samples was measured using Western blotting. All data were obtained from at least three independent experiments and are shown as the means ± S.D., *P<0.05, **P<0.01, ***P<0.001.

To investigate whether APN participates in the liver I/R process, we measured serum APN concentrations using ELISA at different time points post-reperfusion. We observed that the APN level was decreased significantly at 3h, 6h, and 12h after I/R in comparison to the non-ischemia group (3h: 7.65±1.10 versus 11.46±1.05 ug/ml, P<0.01; 6h: 8.14±0.71 versus 11.46±1.05 ug/ml, P<0.001; 12h: 8.93±1.16 versus 11.46±1.05 ug/ml, P<0.05). At 24h, the serum APN level recovered to almost the normal range. However, no change in expression of APN in liver tissue during I/R was seen. The serum APN concentration and APN expression in the liver are shown in Figure 1.

### APN alleviates hepatocyte injury and reduces histopathologic disorder

**Figure 2:**
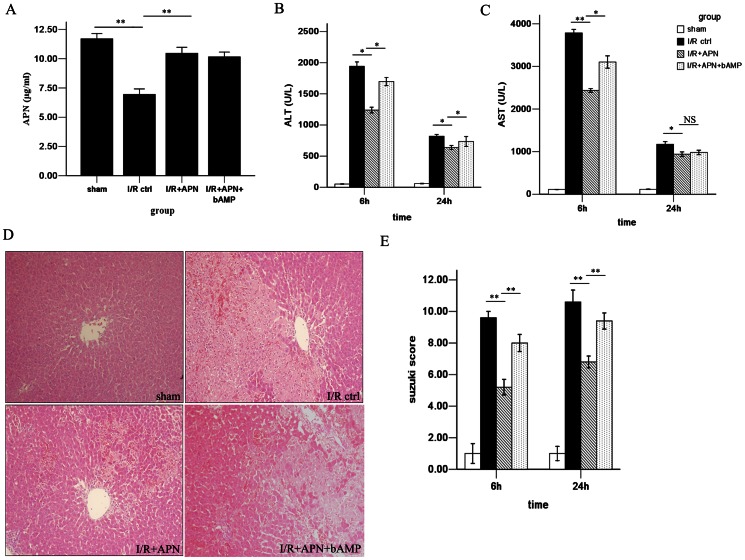
Exogenous APN increases circulatory APN level and improves liver dysfunction during I/R. (A) Serum APN concentration was increased after APN injection. (B) Serum ALT and (C) AST levels in the sham group, I/R control group I/R+APN group and I/R+APN+bAMP group. (D) Representative photomicrograph of liver histology in the sham group, I/R control group I/R+APN group and I/R+APN+bAMP group at 24h after reperfusion. (E) Suzuki scores were presented in the sham group, I/R control group, I/R+APN group and I/R+APN+bAMP group.

To evaluate the role of APN in liver I/R injury, purified APN protein was infused immediately after reperfusion. This infusion led to an increase in circulatory APN levels in the I/R+APN group when compared to the I/R control group (10.46±0.7 versus 7.73±1.04 ug/ml, P<0.01) (Figure 2A). To assess hepatocyte injury, we tested liver function after I/R. In the I/R+APN group, the serum ALT and AST levels were significantly lower than in the I/R control group, especially at 6h after I/R (ALT: 1239.2±102.2 versus 1942.4±154.4 U/L, P<0.05; AST: 2436.8±200.1 versus 3785.2±183.5 U/L, P<0.01) (Figure 2B and 2C). In addition to liver function, the Suzuki histological grading was used to assess hepatocellular damage. Indeed, less lobular edema, congestion, ballooning and hepatocellular necrosis were observed in the I/R+APN group when compared with the control group at 24h after reperfusion (Suzuki score 7.40±0.55 versus 10.72±0.83, P<0.01) (Figure 2D and 2E).

### The effects of APN on inflammatory cell infiltration

**Figure 3:**
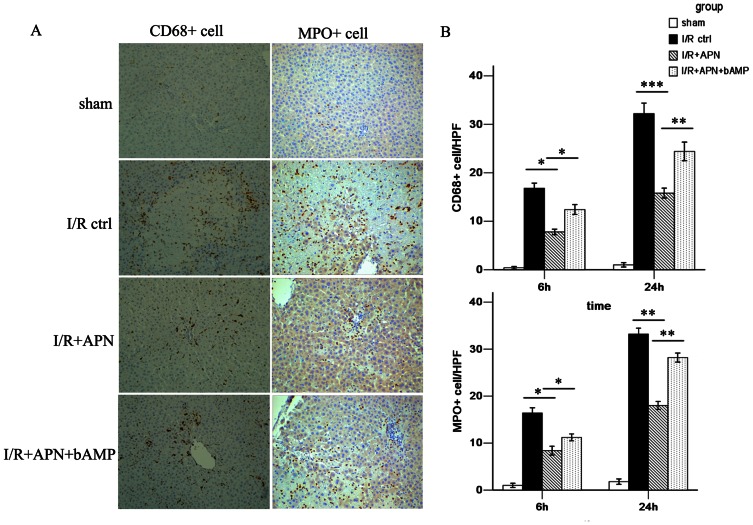
APN inhibits macrophage and neutrophil infiltration. (A) Representative immunohistochemical staining showing CD68^+^ macrophages and MPO^+^ neutrophils in the sham group, I/R control group, I/R+APN group and I/R+APN+bAMP group at 24h after reperfusion. (B) Quantitative analysis of CD68^+^ macrophages and MPO+ neutrophils per high power field (HPF) in each experimental group.

The inflammation response is of paramount importance during liver I/R injury. Macrophages initiate the early phase of injury and promote production and release of proinflammatory cytokines. In the later phase, neutrophils directly cause hepatocyte damage by releasing oxidants and proteases[21]. In this study, inflammatory cell infiltration was measured using CD68 (a marker for macrophages) and MPO (a marker for neutrophils) staining (Figure 3A). The immunohistochemical image showed that less macrophage infiltration was present in the liver of the I/R+APN group (24h: 15.8±1.0/HPF versus 32.3±4.9/HPF, P<0.001). Neutrophil infiltration was also reduced after APN treatment (24h: 18.8±3.2/HPF versus 34.7±6.1/HPF, P<0.01, Figure 3B).

### The effects of APN on pro-inflammatory chemokine and cytokine production

**Figure 4:**
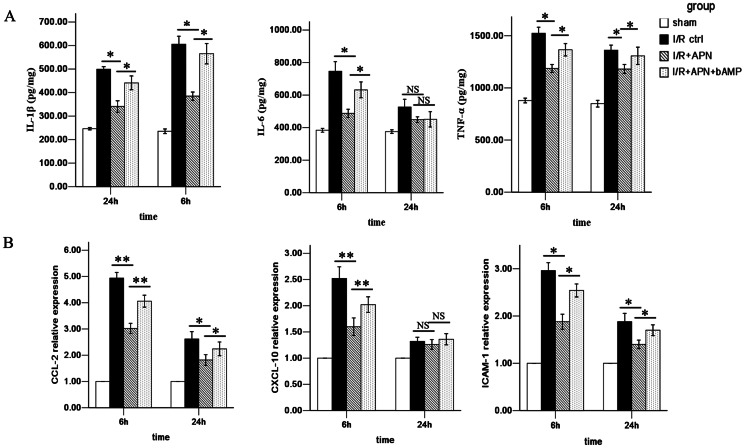
APN reduces pro-inflammatory cytokines/chemokines release in injured liver. (A) IL-1β, IL-6 and TNF-α were detected by ELISA in liver tissue of the sham group, I/R control group, I/R+APN group and I/R+APN+bAMP group.. (B) CCL-2, CXCL-10 and ICAM-1 were detected by RT-PCR in each experimental group.

Pro-inflammatory chemokines including CCL-2, CXCL-10, ICAM-1 and cytokines including IL-1β, IL-6, and TNF-α are important factors regulating initiation and propagation of inflammatory injury during liver I/R[22], [23]. One of the important effects of these chemokines/cytokines is to amplify the deleterious effects of neutrophils and CD4^+^ T lymphocytes on hepatocytes[24]. We found that the levels of all these cytokines/chemokines were lower in the I/R + APN group in comparison to the I/R control group at 6h (IL-1β: 385.0±38.9 versus 605.4±75.1 pg/mg, P<0.05; IL-6: 487.6±55.6 versus 746.5±132.5 pg/mg, P<0.05; TNF-α: 1186.6±84.1 versus 1523.5±129.2 pg/mg, P<0.05; CCL-2: 3.02±0.43 versus 4.94±0.46, P<0.01; CXCL-10: 1.60±0.37 versus 2.52±0.49, P<0.01; ICAM-1: 1.88±0.36 versus 2.96±0.38, P<0.05) (Figure 4), indicating an immunoregulatory effect of APN on inflammatory factors.

### APN alleviates I/R-induced hepatocyte apoptosis

**Figure 5:**
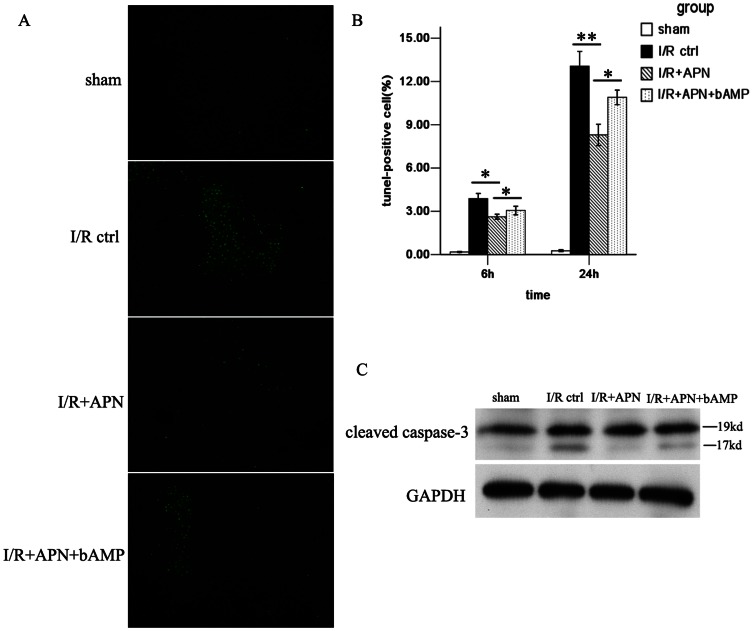
Hepatocyte apoptosis was diminished by APN treatment. (A) Representative liver sections for TUNEL staining of apoptotic cells (green cells) from each experimental group were shown at 24h after reperfusion. (B) Quantitative analysis of TUNEL-positive hepatocyte nuclei per total nuclei in each experimental group is presented. (C) expression levels of cleaved caspase-3, in the liver in each experimental group at 24h after reperfusion are shown.

Cell apoptosis is another effect of I/R injury and can serve as a measure of the extent of I/R injury. In this study, we found that APN treatment alleviated hepatocyte apoptosis during I/R, evidenced by reduced amounts of TUNEL positive cells (8.3±1.6% versus 13.1±2.3%, P<0.01) (Figure 5A and Figure 5B) and less caspase-3 expression in the liver (Figure 5C), especially at 24h post-reperfusion.

### APN activates the AMPK/eNOS pathway

**Figure 6:**
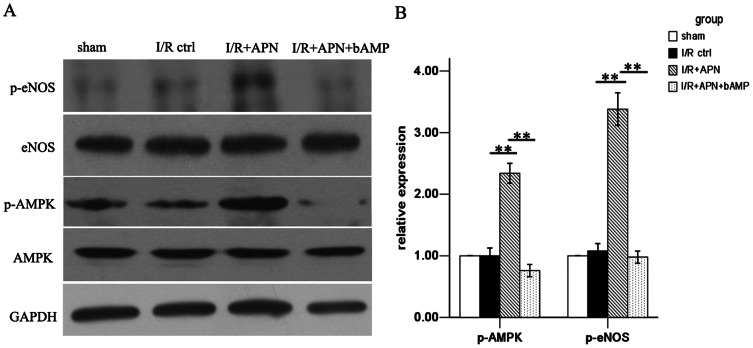
AMPK/eNOS pathway was activated in vivo by APN. (A) Phosphorylation of AMPK at Thr172 and phosphorylation of eNOS at Ser1177 was increased in I/R+APN group at 24h after reperfusion. (B) Quantitative analysis of phosphorylated AMPK and eNOS expression are presented.

After confirming the role of APN in liver I/R, we next tried to determine the underlying mechanism of APN in liver I/R protection. A number of pathways involving molecules like AMPK, AKT, and STAT-3 have been reported to be associated with APN-mediated effects[25], [26]. Herein, we found that APN activates AMPK via increasing phosphorylation of AMPK at Thr172, as well as phosphorylation of its downstream molecule, eNOS at Ser1177 (Figure 6). To understand whether the beneficial effects of APN on liver I/R was AMPK dependent, we inhibited AMPK activation using an AMPK inhibitor, bAMP. After blocking the AMPK pathway, we found that the protective effect of APN was partly abrogated, evidenced by increased aminotransferase levels (Figure 2B and 2C), higher Suzuki score (Figure 2E), and more profound inflammatory cell infiltration (Figure 3), increased proinflammatory chemokines/cytokines levels (Figure 4), and more hepatocyte apoptosis (Figure 5).

### Therapeutic window of APN after reperfusion

**Figure 7:**
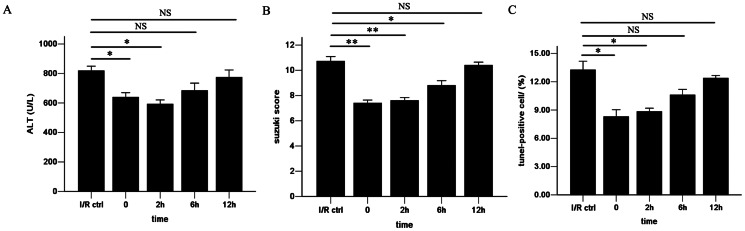
Therapeutic window of APN after reperfusion.

To investigate the potential clinical value of APN, we tried to determine the therapeutic window of APN during reperfusion phase of I/R. In addition to the above experiments in which APN was infused 0h after reperfusion, administration of APN at different time points was performed. The results revealed that injection at 0h and 2h after reperfusion have equal benefit on I/R injury, as serum ALT, Suzuki score and hepatocyte apoptosis were reduced as shown in Figure 7. However, use of APN at 6h or 12h after reperfusion had very little therapeutic value.

## Discussion

Reperfusion after ischemia enhances liver damage during liver transplantation and other surgical procedures. Although efforts have been made, few effective pharmacologic treatments are available in clinical practice. In the current study, we found that APN could protect the liver against inflammatory injury and hepatocyte apoptosis during I/R. To our knowledge, this was the first study demonstrating the protective role of APN in non-fatty liver I/R injury.

APN is emerging as a protein hormone with multiple properties. Since it is derived from adipocytes, the majority of studies focused on obesity-related diseases like fatty liver. Renata Belfort et al. reported that the serum concentration of APN was lower in patients with nonalcoholic steatohepatitis[27]. And they found that increased release of APN was one of the mechanisms through which pioglitazone treats steatohepatitis. Their study indicates that APN is involved in chronic liver disease. In this study, we showed that serum APN was also decreased after acute liver I/R. In addition, other investigators found that the circulatory APN level was inversely correlated with the severity of renal I/R injury[28]. Based on these findings, we hypothesized that exogenous administration of APN might protect the liver from I/R injury. We confirmed this hypothesis by showing decreased aminotransferase levels and amelioration of histopathologic injury when rats were treated with APN during liver I/R injury.

APN is mainly produced by adipose cells and released into the circulation. In recent years, there has been some controversy about whether hepatocytes themselves can synthesize APN. Some people believe that APN is taken up by hepatocytes from the circulation[29], [30], while some suggest the liver can also produce APN[31]. In a study concerning warm ischemia in fatty liver, APN expression in the liver tissue was found to be increased following I/R[32], but circulating APN was not increased in the same way. The lack of a correlation between circulating and liver APN levels led them to think of the possibility of liver-derived APN. However, they also realized the possibility that steatotic hepatocytes took up APN from the circulation. In contrast to their results, we found that the circulatory APN level, rather than the APN expression in nonfatty liver was altered during non-fatty liver I/R. Plasma APN levels are reduced in individuals with obesity, steatohepatitis, type 2 diabetes and coronary artery disease, all traits with chronic inflammation[7]. In the current model, which was an acute inflammation model, the main source of APN, circulatory APN, was also reduced.

It is well-documented that inflammation and cell apoptosis are two major mechanisms of I/R injury. In I/R-mediated inflammation, macrophages initiate the I/R response, followed by the release of pro-inflammatory cytokines/chemokines and neutrophil recruitment under I/R stress[24]. Early stage cytokines including TNF-α and IL-1β are produced by macrophages, while IL-6 could be produced by macrophages, neutrophils or hepatocytes. During the I/R-induced inflammatory response, macrophage activation plays a central role, through inducing neutrophil transmigration from the vascular lumen into the hepatic interstitium via over-expressing various adhesion molecules and chemokines, for instance, ICAM-1, CCL-2 and CXCL-10[21]. On the other hand, the anti-inflammatory activity of APN has been reported in numerous conditions including organ I/R injury. Rei Shibata et al. demonstrated that APN prevented TNF-α production and limited inflammatory injury in a heart I/R injury model[11], while APN infusion also protected kidneys against inflammation and apoptosis during kidney I/R injury[28]. In our experiments, we showed less macrophage and neutrophil infiltration, decreased expression of pro-inflammatory chemokines and adhesion molecule like CCL-2, CXCL-10, ICAM-1 and decreased expression of the cytokines IL-1β, IL-6, and TNF-α during liver I/R injury after treatment with APN. These results demonstrate that APN can also exhibit protective effects during liver I/R injury through inhibiting the inflammatory cascade, from the initial macrophage activation and chemokines/cytokines release, to the later phase of neutrophil recruitment. Because APN functions through inhibiting macrophage activation and chemokines/cytokines at the very beginning after reperfusion, administration of APN at later time points after reperfusion (6h and 12h) probably has little therapeutic value as shown by out data.

Necrosis is caused by acute metabolic perturbation with ATP depletion during liver I/R. Apoptosis, in contrast, represents the execution of an ATP dependent death program often initiated by death ligand/death receptor interactions, such as Fas ligand with Fas, which leads to a caspase activation cascade[33]. A number of studies were focused on I/R-induced hepatocyte apoptosis and they showed that more apoptosis happened in the late phase of I/R[34]. Although APN has been shown to have anti-apoptotic effects, the effect of APN on I/R-induced apoptosis is unclear. In the present study, decreased caspase-3 expression and less apoptotic hepatocytes were observed under treatment with APN. These data indicate that APN can prevent I/R-induced hepatocyte apoptosis. Since TNF-α was a strong stimuli to initiate hepatocyte apoptosis during I/R[2], the anti-apoptotic effects of APN might result from inhibition of TNF-α release.

Distinct mechanisms are involved in the function of APN during different pathophysiological processes. The protective effect of APN on heart I/R injury was found to be dependent on AMPK and COX-2 pathways, while PPARα was induced in I/R-exposed kidneys treated with APN. In the present study, AMPK activation by APN was observed during liver I/R. Blockade of the AMPK pathway partially reversed APN’s protective effect during liver I/R. Importantly, AMPK activation has been shown to play a critical role in reducing liver I/R injury. Carmen Peralta et al. showed that an AMPK activator, AICAR, was useful in reducing liver I/R injury[5]. Additionally, Hjalmar R. Bouma summarized all available literature regarding the protective signaling pathways activated by I/R and concluded that AMPK activation before or during organ preservation might be a promising pharmacologic approach to limit organ injury and maintain graft quality before transplantation[35]. eNOS is an downstream factor in the APN-AMPK pathway and it could promote nitric oxide (NO) generation after activation, which has been shown to be a beneficial vasodilator during I/R[7]. Previous study have also shown that activation of eNOS could ameliorate liver I/R[36]. In our study, eNOS was also activated after APN treatment. All these findings further suggest a critical role of the APN-AMPK-eNOS pathway in liver I/R injury. It is of interest to investigate the detailed mechanisms by which APN regulates the AMPK pathway in the future.

In conclusion, we have demonstrated that serum APN levels fall in response to liver I/R. In addition, our results suggest that infusion of recombinant APN may benefit non-fatty livers subjected to I/R injury by reducing the inflammatory response and hepatocyte apoptosis. The protective effects of APN infusion during liver I/R likely involve the AMPK/eNOS pathway. This study provides a novel treatment target for the protection of livers from I/R injury.
